# Prevalence of aortic aneurysms and dissections in patients with systemic vessel aneurysms and dissections; matched case-control study using a national sample cohort

**DOI:** 10.3389/fcvm.2023.1266430

**Published:** 2023-10-23

**Authors:** Jihye Song, Yong Cheol Lim, Do Jung Kim

**Affiliations:** ^1^ Department of Neurosurgery, Ajou University School of Medicine, Ajou University Hospital, Suwon, Republic of Korea; ^2^ Department of Thoracic and Cardiovascular Surgery, Ajou University School of Medicine, Ajou University Hospital, Suwon, Republic of Korea

**Keywords:** aneurysm, aortic disease, aortic dissection, aortic aneurysm, peripheral artery disease

## Abstract

**Objective:**

Aneurysms in systemic arteries are rare, and little is known about their relationship with aortic aneurysms. In this study, we aimed to evaluate the prevalence of aortic aneurysms and dissections (AAD) in patients with other systemic vessel aneurysms and dissections (OVAD) and identify their potential risk factors.

**Methods:**

This cross-sectional study used a nationwide representative cohort sample from the Korea National Health Insurance Service-National Sample Cohort database. We defined OVAD as systemic vessel aneurysms and dissections excluding intracranial and aortic dissections and aneurysms. With a total of 690 OVAD patients and 2,760 non-OVAD matched controls, we investigated the prevalence of AAD in patients with OVAD and potential risk factors for their concurrence using the *χ*^2^ test and logistic regression.

**Results:**

The prevalence of AAD in patients with OVAD was 10.6% (73/690) and 0.3% (9/2,760) in patients with non-OVAD. The adjusted odds ratio (OR) for having concurrent AAD with OVAD was 37.56 (95% CI: 18.29–77.12, *p* < 0.001) after stratification by sex, age, income, region of residence and after adjustment for hypertension, diabetes mellitus, dyslipidemia, and extent of disability. The adjusted ORs of AAD were significantly higher in females [adjusted OR = 47.63 (95% CI: 10.72–211.55)], and individuals aged ≥60 years [adjusted OR = 28.18 (95% CI: 13.42–59.17)], as well as those without hypertension [adjusted OR = 95.44 (95% CI: 18.21–500.23)], diabetes mellitus [adjusted OR = 46.39 (95% CI: 18.85–114.17)], without dyslipidemia [adjusted OR = 60.99 (95% CI: 20.83–178.56), *p* < 0.001 for all]. The prevalence of AAD significantly differed by according to specific sites of OVAD in carotid artery, upper extremity artery, iliac artery, lower extremity artery, and splanchnic artery (*p* < 0.001 for all).

**Conclusions:**

The prevalence of AAD in patients with OVAD was 37.56 times higher than that in the matched population. We may approach aneurysms as systemic diseases and further investigations of pathophysiology would help to clarify the relationships between AAD and OVAD.

## 1. Introduction

Aneurysms, abnormal dilatations in blood vessels, may be a local manifestation of systemic conditions. Most aneurysms develop through complex processes involving the upregulation of the proteolytic pathways, inflammation, and degradation of the arterial wall matrix. These processes are influenced by systemic and focal factors (1). The natural history of most aneurysms includes an increasing risk of rupture, or in some cases (especially popliteal aneurysms) thrombosis, distal embolization, or both. Aneurysms can manifest in different vessels throughout the body, with various prevalence depending on their location. Some aneurysms in different locations share risk factors, and other aneurysms exist concurrently with rare aneurysms, suggesting a common pathophysiology (2–4).

The overall prevalence of aortic aneurysms and dissections (AAD) were estimated at about 1%–3% in the general population and up to 10% in the older age groups (5–7). This makes it a significant cause of mortality, contributing to approximately 152,000 deaths worldwide in 2013 (8). For abdominal aortic aneurysm (AAA), the mortality rate of ruptured AAA is between 65% and 85% (9). Guidelines have recommended screening for all men at 65 years and all men and women with a true peripheral aneurysm because the mortality rate associated with preventive elective treatment is about 2%–6% (10, 11). On the other hand, aneurysms rarely occur in patients with other systemic vessel aneurysms and dissections (OVAD), including those of splanchnic, renal, or limb arteries and the underlying mechanism remains unclear. Peripheral aneurysms occur most frequently in the popliteal artery with a prevalence of <0.1% (12). These aneurysms are more frequent in older men, reflecting a similar demographic to that of AAD (13). Some studies suggested screening for AAA in patients with popliteal artery aneurysm based on their higher prevalence in patients with popliteal aneurysm (3).

Despite the substantial clinical implications, studies on the coexistence between AAD and OVAD are scarce. This is primarily due to the rarity of OVAD, which results in a limited understanding of the prevalence of specific types of OVAD and their association with AAD. In the present study, we aimed to evaluate the prevalence of AAD in a population-based, large cohort of patients with OVAD and identify potential risk factors for AAD in this population. Understanding the potential association between AAD and OVAD could help understanding shared underlying mechanisms and improving the ability to screen and manage these conditions. It would enhance our perspective of aneurysmal disease as a systemic condition and enable the development of targeted interventions and monitoring strategies for patients at risk.

## 2. Materials and methods

### 2.1. Data collection

This national cohort study relied on data from the Korean Health Insurance Review and Assessment Service-National Sample Cohort (HIRA-NSC). The HIRA-NSC selects samples directly from the entire population database to avoid non-sampling errors. Approximately 2% of the samples (one million) were selected from the entire Korean population (50 million). The selected data were classified into 1,476 levels [age (18 categories), sex (2 categories), and income level (41 categories)] using randomized stratified systematic sampling methods via proportional allocation to represent the entire population. After data selection, the appropriateness of the sample was verified by a statistician who compared the sample data with the data from the entire Korean population. The detailed methods used to perform these procedures are provided by the National Health Insurance Sharing Service. The cohort database included (i) personal information, (ii) health insurance claim codes (procedures and prescriptions), (iii) diagnostic codes using the International Classification of Disease-10 (ICD-10), (iv) death records from the Korean National Statistical Office (using the Korean Standard Classification of disease), (v) socioeconomic data (residence and income), and (vi) medical examination data for each participant over a period ranging from 2002 to 2013 (14–16).

We defined OVAD as systemic vessel aneurysms and dissections excluding intracranial and aortic dissections and aneurysms. The criteria that we employed for extracting the OVAD cohort from the database identified subjects who had been diagnosed at least once with the ICD diagnosis code I72.x and the AAD cohort included subjects who had been diagnosed at least once with ICD diagnosis code I71.x (Table 1). No sample size analyses were done before starting the study.

**Table 1:** Sites of AAD, and OVAD according to their code.

Code	Site	*n* (%)
Total OVAD cases		690/1,025,343 (0.067%)
I72	Other aneurysm and dissection	69 (0.006%)
I72.0	Aneurysm and dissection of the carotid artery	159 (0.016%)
I72.1	Aneurysm and dissection of the artery of the upper extremity	20 (0.002%)
I72.2	Aneurysm and dissection of the renal artery	27 (0.003%)
I72.3	Aneurysm and dissection of the iliac artery	60 (0.006%)
I72.4	Aneurysm and dissection of the artery of the lower extremity	42 (0.004%)
I72.5	Aneurysm and dissection of other precerebral arteries	22 (0.002%)
I72.8	Aneurysm and dissection of the splanchnic artery	137 (0.013%)
I72.9	Aneurysm and dissection of an unspecified site	206 (0.020%)
Total AAD cases		1,546/1,025,343 (0.151%)
I71	Aortic aneurysm and dissection	108 (0.010%)
I71.0	Dissection of the aorta, any part	464 (0.045%)
I71.1	Thoracic aortic aneurysm, ruptured	45 (0.004%)
I71.2	Thoracic aortic aneurysm, without mention of rupture	290 (0.028%)
I71.3	Abdominal aortic aneurysm, ruptured	72 (0.007%)
I71.4	Abdominal aortic aneurysm, without mention of rupture	454 (0.044%)
I71.5	Thoracoabdominal aortic aneurysm, ruptured	23 (0.002%)
I71.6	Thoracoabdominal aortic aneurysm, without mention of rupture	84 (0.008%)
I71.8	Aortic aneurysm of unspecified site, ruptured	45 (0.004%)
I71.9	Aortic aneurysm of unspecified site, without mention of rupture	452 (0.044%)

OVAD, other systemic vessel aneurysms and dissections; AAD, aortic aneurysms and dissections. Subcategories of diagnostic code were included redundantly.

### 2.2. Study cohort and variables

**Figure 1:**
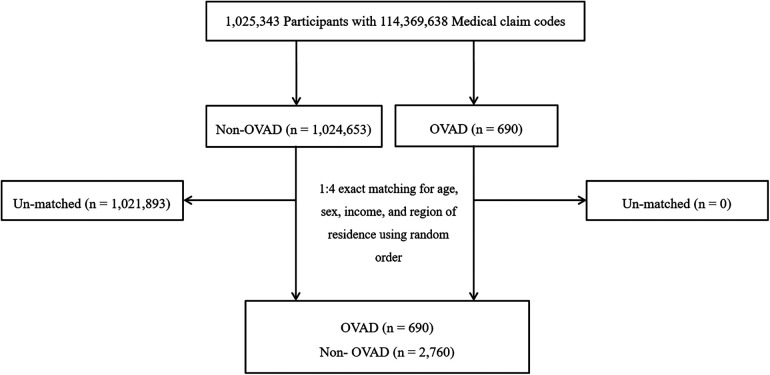
A schematic illustration of the participant selection process that was used in the present study. Of a total of 1,025,343 Participants, 690 of OVAD participants were 1:4 matched with 2,760 non- OVAD participants for age, sex, income, and region of residence. OVAD, other vessel aneurysms and dissections.

OVAD participants were selected from 1,025,343 cases with 114,369,638 medical claim codes from 2002 through 2013 (*n* = 690). The non-OVAD group was included if participants were not defined as OVAD from 2002 through 2013 (*n* = 1,024,653). OVAD participants were 1:4 matched with non-OVAD participants for age, sex, income, and region of residence. To prevent selection bias when selecting the matched participants, the non-OVAD participants were sorted using a random number order and were then selected from top to bottom. It was assumed that the matched non-OVAD participants were being evaluated at the same time as each matched OVAD. The index date refers to the day on which the ICD code occurred. Therefore, the participants in the non-OVAD group who died before the index date were excluded. During matching procedure, 1,021,893 of non-OVAD participants were excluded. Ultimately, 690 of OVAD participants were 1:4 matched with 2,760 non-OVAD participants (Figure 1).

The age groups were classified using the following 10-year age intervals: 0–9, 10–19, …, 70–79, and 80 + years old. A total of 9 age groups were designated. The income groups were initially divided into 41 classes (one health aid class, 20 self-employment health insurance classes, and 20 employment health insurance classes). They were divided into 10 income brackets (deciles) based on their income quintiles and then classified into lower (brackets 1–4), middle (brackets 5–7), or high (brackets 8–10) income tiers. The region of residence was divided into 16 areas according to administrative district. These regions were regrouped into urban (Seoul, Busan, Daegu, Incheon, Gwangju, Daejeon, and Ulsan) and rural (Gyeonggi, Gangwon, Chungcheongbuk, Chungcheongnam, Jeollabuk, Jeollanam, Gyeongsangbuk, Gyeongsangnam, and Jeju) areas. The participants' prior medical histories were evaluated using ICD codes. To ensure an accurate diagnosis, hypertension (I10 and I15), diabetes (E10–E14), and dyslipidemia (E78) were regarded as present if a participant was treated ≥2 time (14, 15, 17). The severity of disability is categorized as 1–6 levels on administrative registration, with a lower number indicating a more severe condition. This study classified people with disability into severe disability (levels 1–2) and mild disability (levels 3–6) groups.

### 2.3. Statistical analysis

A chi-square test was used to compare general characteristics between the OVAD and non-OVAD group (Table 2). A conditional logistic regression model was used to analyses the odds ratio (OR) of OVAD (independent variable) for AAD (dependent variable). Crude (simple) and adjusted (hypertension, diabetes mellitus, dyslipidemia, and extent of disability) models were used in these analyses, and the 95% confidence interval (CI) was calculated. In these analyses, age, sex, income, and region of residence were used as stratification factors. For the subgroup analyses using the conditional logistic regression model, we divided participants by age (<60 years and ≥60 years), sex (men and women), income (low, middle, and high), and region of residence (urban and rural residents). We performed additional subgroup analyses using the conditional logistic regression model, we divided participants by hypertension, diabetes mellitus, dyslipidemia and extent of disability (Table 3). Fisher's exact test was used to compare the prevalence of dependent variable between the OVAD and non-OVAD group (Table 4). Two-tailed analyses were performed, and significance was defined as *p*-values less than 0.05. With Bonferroni correction, we considered *p*-values less than 0.05/9 to account for the increased risk of type I error associated with conducting multiple statistical tests (Table 4). The Statistical Analysis System (SAS) version 9.4 (SAS Institute Inc., Cary, NC, USA) were used for statistical analyses.

**Table 2:** General characteristics of OVAD and non-OVAD participants.

Characteristics	OVAD (*n*, %)	Non-OVAD (*n*, %)	*p*-value
Total participants	690 (100.0)	2,760 (100.0)	
Age (years)			1.000
0–9	3 (0.4)	12 (0.4)	
10–19	3 (0.4)	12 (0.4)	
20–29	19 (2.8)	76 (2.8)	
30–39	33 (4.8)	132 (4.8)	
40–49	108 (15.7)	432 (15.7)	
50–59	176 (25.5)	704 (25.5)	
60–69	182 (26.4)	728 (26.4)	
70–79	138 (20.0)	552 (20.0)	
80+	28 (4.1)	112 (4.1)	
Age group			1.000
<60	342 (49.6)	1,368 (49.6)	
≥60	348 (50.4)	1,392 (50.4)	
Sex			1.000
Male	333 (48.3)	1,332 (48.3)	
Female	357 (51.7)	1,428 (51.7)	
Income			1.000
Low	203 (29.4)	812 (29.4)	
Middle	237 (34.4)	948 (34.4)	
High	250 (36.2)	1,000 (36.2)	
Region of residence			1.000
Urban	357 (51.7)	1,428 (51.7)	
Rural	333 (48.3)	1,332 (48.3)	
Hypertension			<0.001
No	230 (33.3)	1,528 (55.4)	
Yes	460 (66.7)	1,232 (44.6)	
Diabetes mellitus			<0.001
No	479 (69.4)	2,134 (77.3)	
Yes	211 (30.6)	626 (22.7)	
Dyslipidemia			<0.001
No	408 (59.1)	1,944 (70.4)	
Yes	282 (40.9)	816 (29.6)	
Extent of disability			<0.001
Non-disability	561 (81.3)	2,423 (87.8)	
Severe	45 (6.5)	79 (2.9)	
Mild	84 (12.2)	258 (9.4)	
AAD			<0.001
No	617 (89.4)	2,751 (99.7)	
Yes	73 (10.6)	9 (0.3)	

OVAD, other vessel aneurysms and dissections; AAD, aortic aneurysms and dissections. Age of participants was measured by the age at the time of the first visit.

**Table 3:** Crude and adjusted odd ratios of AAD in relation to the status of OVAD.

Variable	Odd ratios for AAD					
	Crude			Adjusted^a^		
	OR	95% CI	*p*-value	OR	95% CI	*p*-value
Total participants						
Non-OVAD	1 (ref)			1 (ref)		
OVAD	36.67	18.20–73.88	<0.001*	37.56	18.29–77.12	<0.001*
Male						
Non-OVAD				1		
OVAD	347.60	15.53–77.10	<0.001*	38.32	16.62–88.37	<0.001*
Female						
Non-OVAD				1		
OVAD	43.66	10.20–186.85	<0.001*	47.63	10.72–211.55	<0.001*
Age <60 years						
Non-OVAD				1		
OVAD	N/A			N/A		
Age ≥60 years old						
Non-OVAD				1		
OVAD	26.67	12.98–54.78	<0.001*	28.18	13.42–59.17	<0.001*
Low income						
Non-OVAD				1		
OVAD	20.34	6.75–61.29	<0.001*	17.88	5.64–56.72	<0.001*
Middle income						
Non-OVAD				1		
OVAD	66.97	15.70–285.67	<0.001*	90.52	19.43–421.68	<0.001*
High income						
Non-OVAD				1		
OVAD	38.54	11.62–127.78	<0.001*	35.80	10.56–121.33	<0.001*
Urban resident						
Non-OVAD				1		
OVAD	59.19	17.94–195.27	<0.001*	71.85	20.52–251.57	<0.001*
Rural resident						
Non-OVAD				1		
OVAD	25.94	10.90–61.73	<0.001*	26.65	10.91–65.01	<0.001*
Non-hypertension						
Non-OVAD				1		
OVAD	70.81	15.78–317.81	<0.001*	95.44	18.21–500.23	<0.001*
Hypertension						
Non-OVAD				1		
OVAD	26.16	11.12–61.55	<0.001*	27.14	11.48–64.20	<0.001*
Non-diabetes mellitus						
Non-OVAD				1		
OVAD	41.96	17.73–99.32	<0.001*	46.39	18.85–114.17	<0.001*
Diabetes mellitus						
Non-OVAD				1		
OVAD	24.23	7.06–83.18	<0.001*	34.94	8.69–140.47	<0.001*
Non-dyslipidemia						
Non-OVAD				1		
OVAD	51.32	18.25–144.29	<0.001*	60.99	20.83–178.56	<0.001*
Dyslipidemia						
Non-OVAD				1		
OVAD	19.99	7.57–52.77	<0.001*	19.72	7.31–53.14	<0.001*
Non-disability						
Non-OVAD				1		
OVAD	44.21	18.85–103.67	<0.001*	46.68	19.47–111.90	<0.001*
Severe disability						
Non-OVAD				1		
OVAD	N/A			N/A		
Mild disability						
Non-OVAD				1		
OVAD	13.51	2.76–66.04	0.001*	25.65	2.74–239.73	<0.001*

OR, odds ratio; CI, confidence interval; AAD, aortic aneurysms and dissections; OVAD, other vessel aneurysms and dissections; ref, reference; N/A, not available.

^a^ Adjusted models were stratified by age, sex, income, and region of residence and adjusted for hypertension, diabetes mellitus, dyslipidemia, and extent of disability.

* Conditional and unconditional logistic regression model, significance at *p* < 0.05.

**Table 4:** Prevalence of AAD according to the specific sites of OVAD (2002–2013).

OVAD		Non-AAD cases (*n* = 1,023,797) (*n*, %)	AAD cases (*n* = 1,546) (*n*, %)	*p*-value
I72	Aneurysm and dissection	617 (0.06)	73 (4.72)	<0.001*
I72.0	Aneurysm and dissection of the carotid artery	155 (0.02)	4 (0.26)	<0.001*
I72.1	Aneurysm and dissection of the artery of the upper extremity	20 (0.00)	0 (0.00)	<0.001*
I72.2	Aneurysm and dissection of the renal artery	22 (0.00)	5 (0.32)	0.970
I72.3	Aneurysm and dissection of the iliac artery	33 (0.00)	27 (1.75)	<0.001*
I72.4	Aneurysm and dissection of the artery of the lower extremity	36 (0.00)	6 (0.39)	<0.001*
I72.5	Aneurysm and dissection of other precerebral arteries	22 (0.00)	0 (0.00)	0.967
I72.8	Aneurysm and dissection of the splanchnic artery	121 (0.01)	16 (1.03)	<0.001*
I72.9	Aneurysm and dissection of an unspecified site	187 (0.02)	19 (1.23)	<0.001*

AAD, aortic aneurysms and dissections; OVAD, other vessel aneurysms and dissections; Subcategories of OVAD diagnostic code were included redundantly.

* Fisher's exact test. Significance at *p* < 0.05/9 for Bonferroni correction.

### 2.4. Ethics

This study adhered to the tenants of the Declaration of Helsinki and was approved by the Institutional Review Board of Ajou University School of Medicine (institutional review board number: AJIRB-MED-EXP-19-544). All analyses adhered to the guidelines and regulations of the ethics committee of Ajou University School of Medicine. The requirement for written informed consent was waived because the Korea National Health Insurance Service-National Sample Cohort data set consists of de-identified secondary data for research purposes.

## 3. Results

### 3.1. Baseline characteristics

The detailed code and numbers for OVAD and AAD are described in Table 1. For the entire cohort, the baseline prevalences of OVAD and AAD were 0.067% (690/1,025,343) and 0.151% (1,546/1,025,343), respectively.

Table 2 presents the general characteristics of OVAD and non-OVAD participants, matched for age, sex, income, and region of residence. A total of 690 OVAD and 2,760 non-OVAD participants were included in the study. The age distribution; sex ratio; the proportions of low, middle, and high-income participants; and the proportion of urban and rural residents were comparable between two groups. However, the prevalence of AAD was significantly higher in the OVAD group (10.6%) compared to non-OVAD group (0.3%, *p* < 0.001). And the OVAD group had significantly higher rates of hypertension (66.7% vs. 44.6%), diabetes mellitus (30.6% vs. 22.7%), and dyslipidemia (40.9% vs. 29.6%) than the non-OVAD group (*p* < 0.001 for all). In addition, the extent of disability was higher in the OVAD group, with a higher proportion of participants with severe (6.5% vs. 2.9%) and mild (12.2% vs. 9.4%) disabilities. These findings suggest that patients with OVAD have a higher prevalence of comorbidities and disabilities, as well as a higher likelihood of having AAD compared to non-OVAD participants.

### 3.2. Prevalence of aortic aneurysm and dissection

Table 3 presents the crude and adjusted OR of AAD in relation to the status of OVAD. The analysis was stratified by factors such as sex, age, income, region of residence and adjusted for hypertension, diabetes mellitus, dyslipidemia, and extent of disability. Our analysis demonstrated a significant association between the presence of OVAD and the occurrence of AAD. The adjusted OR for AAD in the total participants was 37.56 (95% CI: 18.29–77.12, *p* < 0.001) in comparison to non-OVAD indicating that the patients with OVAD were approximately 37.56 times more likely to have AAD than those without OVAD. In the stratified analysis, the adjusted ORs of AAD were notably higher among females (adjusted OR = 47.63), individuals aged ≥60 years (adjusted OR = 28.18), middle-income earners (adjusted OR = 90.52), and urban residents (adjusted OR = 71.85, *p* < 0.001 for all). The adjusted ORs of AAD were higher in patients without hypertension (adjusted OR = 95.44), diabetes mellitus (adjusted OR = 46.39), dyslipidemia (adjusted OR = 60.99), and disability (adjusted OR = 46.48, *p* < 0.001 for all). These findings suggest the association between these confounders and an increased likelihood of AAD in patients with OVAD even after stratification and adjustment for several confounders.

Table 4 presents the prevalence of AAD according to specific sites of OVAD and compares the prevalence of AAD participants (*n* = 1,546) and non-AAD participants (*n* = 1,023,797) across various diagnostic codes (I72.0–I72.9). A significant difference in prevalence was observed between AAD and non-AAD participants in several OVAD subcategories. The prevalence of OVAD of carotid artery (I72.0) was 0.02% (*n* = 155) in non-AAD participants, whereas the AAD participants demonstrated a higher prevalence of 0.26% (*n* = 4, *p* < 0.001). Similar significant differences were observed in other subcategories; upper extremity artery, iliac artery, lower extremity artery, and splanchnic artery (*p* < 0.001 for all). However, no significant differences were found for renal artery (*p* = 0.970) and other precerebral arteries (*p* = 0.967). These findings suggest that the presence of AAD varies substantially depending on the specific site of OVAD.

## 4. Discussion

The present study demonstrated that the prevalence of AAD in patients with OVAD was 37.56 times higher than that in the matched population. OVAD patients without known cardiovascular risk factors, such as hypertension, diabetes mellitus, and dyslipidemia showed an increased OR after stratification by age, sex, income, and region of residence and after adjustment for hypertension, diabetes mellitus, dyslipidemia, and extent of disability. This implies that these subgroups had an even higher likelihood of coexisting AAD. This is the first population-based study to investigate the prevalence of AAD in patients with OVAD.

There are few reports about the prevalence of AAD in patients with OVAD because the rarity of most OVAD, such as those affecting splenic or pulmonary arteries, upper limb arteries. Consequently, little is known about their prevalence, and they do not seem to be associated with the more common aneurysms observed in other locations (1). The coexistence of AAD and OVAD has been partially reported in previous studies. Among peripheral aneurysms, popliteal artery aneurysms are the most common, with a prevalence of <0.1% (12). They are common in individuals in their 70s and in men, which is a characteristic similar to that of an aortic aneurysm (13). Patients with popliteal artery aneurysms have a higher risk of concurrent peripheral and aortic aneurysms (4). Approximately 64% of these patients have contralateral popliteal artery aneurysms. Moreover, 40%–60% of cases present with a simultaneous abdominal aortic aneurysm, and this prevalence further rises to 70% when bilateral popliteal aneurysms are present (18–21). The femoral artery is another common site for concurrent aneurysms, in nearly 40% of cases (20). Conversely, a large-scale population screening study reported that 25% of AAAs have coexisting common iliac arteries, and in 7% of these patients have concurrent internal iliac artery aneurysms (22). Another study with AAA patients revealed a 14% incidence of popliteal artery aneurysm (23). In an autopsy study of 205 cases of thoracic aortic aneurysm, 43% of men and 26% of women with a thoracic aortic aneurysm had concurrent abdominal aortic, iliac, or femoral aneurysms (5). Patients with AAAs show mild dilation and reduced distensibility in their carotid arteries compared to controls (24, 25).

Previous reports also showed the coexistence of aneurysms in different locations, suggesting a common pathophysiology. Recent systemic review demonstrated that one in six patients with a primary aneurysm harbors a concurrent aneurysm, and one in four if the patient has a popliteal aneurysm (4). In our previous population-based study, the prevalence of intracranial aneurysm in patients with OVAD was 25.7%, which is approximately 20 times higher than that in the general population. We also showed the prevalence of intracranial aneurysm in patients with AAD was 6.8%, which is approximately 4 times higher than that in the general population (14). Another study demonstrated an increased prevalence of extracranial carotid artery aneurysms in patients with intracranial aneurysm (26). They showed that the prevalence of these aneurysms in patients with intracranial aneurysm was 2%, which is higher than that in the general population (<1%) (27, 28). Intracranial aneurysms showed a higher prevalence in patients with other vasculopathies, such as cervicocephalic arteriopathies (15, 29), bicuspid aortic valve, coarctation of the aorta (30), aortic aneurysm (14, 29), and dissection (14, 29), and in patients with systemic vessel aneurysms (15). About 7% of patients with AAAs and 5% with thoracic aortic aneurysms have an intracranial aneurysm (2, 5). Previous reports demonstrated that patients with aneurysmal disease often exhibit abnormalities throughout their entire vascular tree. For instance, AAA has been reported to be associated with generalized arteriomegaly (22). Changes in the matrix composition found in AAA walls have been observed in non-aneurysmal aortic segments and the inferior mesenteric vein (31, 32). Additionally, patients with coronary artery ectasia often experience varicose (33). The investigation of etiology and pathogenesis in aneurysmal disease is particularly focused on patients with multiple aneurysms, as they may provide insights into the underlying mechanisms. Only minority of patients with aneurysms have identifiable pathological causes, while the underlying mechanisms for most aneurysms are unknown and debated. Both systemic and focal factors may play a role in their pathogenesis. Aneurysms in other systemic vessels are rare, with limited knowledge about their prevalence or pathophysiology. In the present study, we observed that the adjusted OR of AAD is higher in females and those without hypertension. Genetic predisposition that influences the structural integrity of the vascular system or metabolic factors might be plausible explanations (34). Also, it may imply that certain hormonal fluctuations in females especially those related to estrogen that have been implicated in various vascular conditions might predispose them to a higher risk of AAD (35, 36).

There is no specific recommendation for the screening of AAD in patients with OVAD. The rupture of an AAA is a life-threatening event that is associated with substantial morbidity and mortality. Preventive treatment-related mortality of unruptured aortic aneurysm is relatively low (10). Identifying the increased likelihood of concurrent aneurysm elsewhere in those with OVAD plays an essential role in the appropriate care of this patient population. This study showed that the prevalence of AAD in patients with OVAD was 37.56 times higher than that in the matched population. It might imply that there would be a remaining large component of unmeasured determinants, such as genetic factors or other metabolic risk factors. Efforts to investigate aneurysmal disease as a systemic disease and further epidemiologic and pathologic studies are needed. The present study also demonstrated that the presence of AAD varies substantially depending on the specific site of OVAD, emphasizing the importance of considering the site of OVAD when assessing the risk of AAD in patients. This information might be helpful for clinicians when evaluating patients with OVAD, screening them identify individuals with a higher likelihood of developing AAD and enabling better-targeted interventions and monitoring strategies.

Our study has several potential limitations. First, we were unable to investigate lifestyle risk factors, such as body mass index, smoking status, sleep, and alcohol consumption because this information was not available in the HIRA-NCS database. Other vascular risk factors, such as chronic kidney disease, chronic inflammation, and family history were not included. Therefore, these possible confounding factors could not be controlled and statistical matching techniques, such as propensity score matching to address these concerns were not applied in this study. The lack of data and analysis is a major limitation of this study. Further studies using medical database established for research purpose are needed. Second, the absence of information on disease severity and subtype limited the possibility of a more detailed analysis, which would have enabled the authors to suggest a more intricate disease mechanism. Third, the observed prevalence of OVAD compared to AAD is higher than previous reports. While this study was based on a large population-based cohort, ensuring ample statistical power and reduced selection bias compared to retrospective reviews of hospital records, it may still have been susceptible to accessibility bias and coding practices by each physician. Therefore, it should be cautious to interpretate our findings. Fourth, the diagnoses of AAD, OVAD, hypertension, diabetes mellitus, and dyslipidemia were based on only the ICD code, which might be less accurate compared to the diagnoses based on medical record data, including medical history, evaluated images, and prescribed drugs. Fifths, we did not exclude the pediatric population. Pediatric OVAD is an extraordinarily rare and completely different spectrum of disease. It might interfere with interpreting accurate results. Sixths, this study lacked the analysis according to the different location and pathology. The association of iliac or popliteal artery aneurysms and AAA is well known and it might affect the result of this study. Lastly, the cross-sectional design of the study implies that these new findings were based on the assumption that AAD precedes OVAD in onset. Verifying the validity of this assumption is challenging due to varying prevalence and onset of OVAD reported in different studies.

In conclusion, aneurysms may be a focal manifestation of systemic conditions. This is the first prevalence study of AAD in patients with OVAD. This nationwide, population-based study found that the prevalence of AAD in patients with OVAD is 10.6% and the adjusted OR was 37.56 times higher than that in the matched population. However, sparse knowledge on the optimal screening of AAD is provided to patients with OVAD. Physicians approach aneurysms as a systemic disease. In addition, further investigations regarding the epidemiology and pathophysiology of AAD in OVAD are required to yield conclusive results.

## Data Availability

Publicly available datasets were analyzed in this study. This data can be found here: All data is available from the database of Korea national health insurance sharing service (https://nhiss.nhis.or.kr). KNHISS does not allow researchers to provide data to other sites personally. Therefore, the authors do not have the right to provide materials to another person or institution. In order to access the original data of this paper, you can follow the KNHISS guidelines and promise to follow the research ethics through the website, and then provide a certain fee and request the raw data. This process requires IRB approval. Everyone can apply for the data at the HIRA-NPS web site (http://nhiss.nhis.or.kr/, tel: +82-33-736-2430, 2431). These processes are intended to get consent from all researchers for the compliance of ethical guidelines not to impede the data sharing. The authors did not have special access privileges to these data sets.
